# Quantification of multiple infections of *Plasmodium falciparum in vitro*

**DOI:** 10.1186/1475-2875-11-180

**Published:** 2012-05-30

**Authors:** Mark A Wacker, Lindsey B Turnbull, Leah A Walker, Michael C Mount, Michael T Ferdig

**Affiliations:** 1 Eck Institute of Global Health, Department of Biological Sciences, University of Notre Dame, Notre Dame, IN, USA; 2 Division of Infection and Immunity, University College London, London, UK; 3 School of Medicine, Case Western Reserve University, Cleveland, OH, USA

## Abstract

**Background:**

Human malaria infections caused by the parasite *Plasmodium falciparum* often contain more than one genetically distinct parasite. Despite this fact, nearly all studies of multiple strain *P. falciparum* infections have been limited to determining relative densities of each parasite within an infection. In light of this, new methods are needed that can quantify the absolute number of parasites within a single infection.

**Methods:**

A quantitative PCR (qPCR) method was developed to track the dynamic interaction of *P. falciparum* infections containing genetically distinct parasite clones in cultured red blood cells. Allele-specific primers were used to generate a standard curve and to quantify the absolute concentration of parasite DNA within multi-clonal infections. Effects on dynamic growth relationships between parasites under drug pressure were examined by treating mixed cultures of drug sensitive and drug resistant parasites with the anti-malarial drug chloroquine at different dosing schedules.

**Results:**

An absolute quantification method was developed to monitor the dynamics of *P. falciparum* cultures *in vitro.* This method allowed for the observation of competitive suppression, the reduction of parasites numbers due to the presence of another parasite, and competitive release, the improved performance of a parasite after the removal of a competitor. These studies demonstrated that the presence of two parasites led to the reduction in density of at least one parasite. The introduction of drug to a mixed culture containing both a drug resistant and drug sensitive parasites resulted in an increased proportion of the drug resistant parasite. Moreover, following drug treatment, the resistant parasite experienced competitive release by exhibiting a fitness benefit greater than simply surviving drug treatment, due to the removal of competitive suppression by the sensitive parasite.

**Conclusions:**

The newly developed assay allowed for the examination of the dynamics of two distinct clones *in vitro*; both competitive suppression and release were observed. A deeper understanding of the dynamic growth responses of multiple strain *P. falciparum* infections, with and without drug pressure, can improve the understanding of the role of parasite interactions in the spread of drug resistant parasites, perhaps suggesting different treatment strategies.

## Background

Natural parasitic infections are often diverse, containing multiple parasite species and/or distinct genotypes of the same species [1]. Parasites of the *Plasmodium* genus, the cause of human malaria, are no exception as infections of multiple strains or species of parasites have been widely reported [2-7] and may be typical in high transmission regions [7,8]. Growth relationships between parasite types within a single host have significant evolutionary implications for selection of fitness and drug resistance traits that can greatly impact public health [9]. However, surprisingly little is known about the dynamics of multi-clone infections and previous investigations have been limited to the determination of relative amounts of parasites within multi-clonal infections. While relative quantification is informative in determining which parasite is present in greater numbers, the reliance on relative abundance limits the insight that can be gained into the dynamics of parasite growth. For example, relative density measurements do not clarify whether the minority parasite population is expanding, albeit at a slower rate than the majority parasite, or if it is static or declining due to the presence of a second parasite; i.e., a parasite that is competitively suppressed will have not only a lower density than the majority parasite but also a lower density than when grown in single culture. Recently, it has been proposed that within-host ecology can be harnessed in control efforts to stop the spread of drug resistant parasites [9], emphasizing the need for tools and experimental approaches to explore within-host dynamics of *Plasmodium falciparum* infections.

Diversity within *P. falciparum* infections is an area of expanding interest. Genotyping highly variable genes, such as merozoite surface protein 1 (*msp1*), merozoite surface protein 2 (*msp2*) and glutamine rich protein (*glurp*), is the most common method of identifying individual clones within an infection [7]. Other studies used microsatellites (MS) [10] and multiplexed real time PCR [11]. More recently, the complexity of infection has also been investigated with massively parallel pyrosequencing on the 454 sequencing platform, which has the distinct advantage of being able to sequence single strands of DNA; consequently, parasite variants that make up only a small proportion of the overall infection population can be detected. Deep sequencing revealed an even greater complexity of infection, with average of 5.0 more variants identified than using nested PCR based methods utilized by earlier studies [7]. In addition, this study showed an expansion of specific *msp2* genotypes after drug treatment, however, the assay is not able to directly link these genotypes to drug resistance [7]. High throughput sequencing has also been used to calculate multiplicity of infection on small amounts of blood taken directly from patients [12].

Most reports of multiple infections of human malaria have relied on relative quantification methods; however, methods that determine the absolute amount of each parasite strain within an infection have been developed for the rodent malaria model *Plasmodium chabaudi*[13-15]. These studies revealed that the presence of two parasite clones led to the reduction in density of at least one clone, a phenotype termed competitive suppression [16]. Additionally, the introduction of drug pressure to a mixed culture containing drug resistant and drug sensitive parasites resulted in a greater transmission potential of the drug resistant parasite. Another effect of drug treatment is that the resistant parasite experiences competitive release. In this case, the resistant parasite exhibits a fitness benefit greater than simply surviving drug treatment, as it actually expands to fill the niche previously inhabited by the sensitive parasite, due to the removal of competitive suppression by the sensitive parasite. The degree of release is dependent both on the timing of drug treatment and the dose given [17,18]. More alarmingly, when a mixed infection containing both sensitive and resistant parasites was treated with drug, the resistant parasite reached a greater density than if it were growing in a host alone, a phenomenon termed treatment-induced facilitation which has also be observed in humans [6,14,17]. The development of similar quantitative methods for *P. falciparum* will enhance efforts to understand the outcomes of multi-clonal infections. These *in vitro* assays allow for control of some of the many variables influencing competitive interactions that cannot be controlled in the host.

## Methods

### Parasite culture

Parasites used for this study include the CQS parasite clone, HB3, and CQR clones, Dd2 and 7C424. HB3 originated in Central America and is sensitive to most anti-malarial drugs except for low-level resistance to pyrimethamine and sulfadoxine. Dd2 is a multidrug resistant parasite originating from Southeast Asia, with high levels of resistance to all anti-malarial drugs except the 8-aminoquinolines. 7C424 is a CQR progeny of the HB3 × Dd2 cross. Parasites were grown in complete media (CM) [370uM hypoxanthine (Hx) (Sigma), and 25 mM HEPES (Sigma); 0.5% Albumax II (Gibco), 10ug/mL gentamycin (Gibco), and 0.225% sodium bicarbonate (Biosource) in RPMI-1640 medium (Gibco)] at approximately 5% hematocrit (hct) in O^+^ RBC. Cultures were maintained at a constant pH, 7.0-7.5, temperature, 37°C, and atmosphere, 5% CO_2_/5% O_2_/90% N_2_ with daily media changes.

### Competition assays

To quantify the dynamics of a multiple infection of Dd2 and HB3 *in vitro,* a qPCR assay was developed using known sequence polymorphisms in the merozoite surface protein 1 (*msp1*) gene. Parasites were synchronized using 5% D-sorbital and assays were initiated 48 hours later. Assays were initiated at 0.25% parasitaemia and 5% haematocrit for single cultures. Mixed cultures were initiated in triplicate at 0.25% parasitaemia of each parasite (total parasitaemia of 0.5%) and 5% haematocrit. Five mL cultures were maintained in triplicate for five full cell cycles. Media was changed daily and a 300 uL sample was retained and stored at −20°C. DNA was extracted from samples using an E.Z.N.A. kit (Omega biotech) following manufacturer’s instructions. To determine the outcome of the assay *t*-test were performed on the data from the last day of the experiment. Assays were stopped on day 11, near the point at which the culture system could not support the general healthy expansion due to high parasitaemia.

For drug treated competition assays parasites were cultured for 72 hours to establish an infection before constant CQ (50 nM) was initiated and maintained for the duration of the assay. Fifty nM CQ was chosen because it represents a high killing dose (approximately the IC90, IC50 = 27.4nM) for HB3, but does not significantly affect CQR parasite 7C424 (approximately the IC10, IC50 = 327.5nM) [19]. Application of drug treatment was delayed to mimic conditions in a mouse model where competitive release was observed when drug treatment was initiated after an infection had been established [17].

Cultures for the drug treated assays and controls were initiated as described above; 50 nM CQ was added to the media and this concentration was maintained for the duration of the assay. Three treatments were used for drug treated assays: (1) a no-drug control identical to the previous no drug assay; (2) CQ drug treated on day 3 for mixed and single culture controls; and (3) CQ drug treated on day 5 for both mixed and single culture controls. As opposed to earlier assays, drug treated assays were grown for 15 days as both the slow growth of 7C424 and reduced parasites numbers due to drug treatment allowed for additional days before the culture could no longer support the healthy growth of the majority parasite.

### Quantification of parasite density

To determine the density of each parasite, allele specific primers were used and compared to a standard curve generated from known parasitaemias. D-soribtol (fluka) synchronized parasites were used to create the standard curve and DNA collected and extracted using an E.Z.N.A. kit (Omega biotech). Parasitaemia of the culture was determined using flow cytometry using previously described methods [20]. The density of red blood cells was determined using Flow count flourosphere (Beckman Coulter). Using the parasitaemia and red blood cell density, the number of parasite genomes per microlitre was calculated.

Allele specific primers were used to determine the amount of each parasite in a mixed culture. The DNA was analysed using a quantitative PCR assay based on a shared *TaqMan* probe (Applied Biosystem). Primers used for the Dd2 parasites are forward (AAT TGC CAA AAC TAT TAA ATT TAA CAT TGA TAG) and reverse (TGA ACA GAT TTC GTA GGA TCT TGT GA) with a probe targeted towards a polymorphic sequence of the gene *msp1* (6 FAM ACT GAT CCA CTT GAA TTA GMGBNFQ). These primers are also used to amplify 7C424 in drug treated assays as 7C424 inherited the Dd2 allele of *msp1*. Primers used for HB3 are forward (GAA ATT GCC AAA ACT ATT AAA TTT AAT ATT GAT AG) and reverse (GGT TCA GTT GAT TCC TTT GTT TCA AC) with the same probe used for Dd2. Logarithmic standard curves were generated in triplicate for each parasite used to ensure accurate quantitation using 0.0 ηg/μL, 0.02 ηg/μL, 0.2 ηg/μL, 2.0 ηg/μL, 20 ηg/μL, and 200 ηg/μL. Primer specificity was determined by generating additional standard curves with 200 ηg/μL of competition parasite DNA added to each well (Additional file 1).

### Statistics

Statistics were performed using GraphPad Prizm 5.0. To determine significant differences between parasites in competition assay t-tests were performed on densities determined on day 11 for Dd2 v HB3 assays and on day 15 for HB3 v 7C424 assays.

## Results

### Assay validation

To determine that the qPCR assay could accurately detect parasite DNA over a wide range of concentrations experiments were carried out to measure the amplification of known quantities of DNA from each of the three parasites used in competition assays (Dd2, HB3 and 7C424). The assay was able to accurately quantify DNA over a 10,000 fold dynamic range (Additional file 1). In addition, it was determined that DNA from a second parasite clone had no significant impact on the ability to quantify parasite DNA from the parasite of interest (Additional file 1).

### Dd2 competitively suppresses HB3

**Figure 1:**
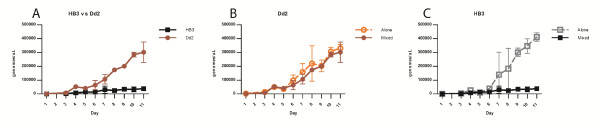
**Infection dynamics of Dd2 and HB3. A)** Dd2 (brown circles) competitively suppresses HB3 (black squares) when parasites are grown together in culture. **B)** No difference is detected between Dd2 grown in single culture (orange circles) and in mixed culture with HB3 (brown circles). **C)** HB3 reaches higher densities when grown in single culture (grey squares) than in mixed culture with Dd2 (black squares). Points are means of three replicates, error bars represent standard deviation.

Pilot data obtained using relative quantification competition assays indicated that when Dd2 and HB3 were co-cultured Dd2 eventually predominated. The growth dynamics were dissected in real time by monitoring co-cultured Dd2 and HB3. Dd2 reached a significantly greater density than HB3 (*t*-test, *p* = 0.0036) (Figure 1A). Dd2 was analysed grown alone as a control and co-cultured with HB3; the presence of HB3 in the mixed culture had no effect on the growth of Dd2 (Figure 1B). However significantly more HB3 was present when grown alone than when co-cultured with Dd2 (*t*-test, *p* < 0.0001), demonstrating that Dd2 competitively suppressed HB3 (Figure 1C). The dynamics of HB3 growth alone and in mixed culture differed greatly. In single culture, HB3 entered an exponential growth phase reaching a maximum average density of 410,814 genomes per microlitre. The maximum HB3 density in mixed culture is 38,682 genomes per microlitre. Further investigation focused on the investigation of the affects of drug treatment on the dynamics of multi-clonal infections.

### Drug treatment alters culture composition

**Figure 2:**
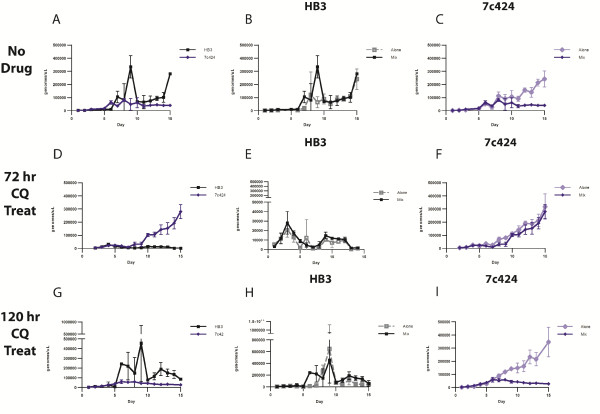
**Infection dynamics of HB3 and 7C424.** In the absence of drug pressure **(A-C)**: A) HB3 (black squares) competitively suppresses 7C424 (blue diamonds). B) No difference is detected betweeHB3 grown in single culture (grey squares) and in mixed culture with 7C424 (closed black squares). C) 7C424 reaches high densities single culture (light blue diamonds) than in mixed culture with HB3 (dark blue diamonds). When drug pressure initiated 72 hours after the start of the assay **(D-F)**: D) 7c424 (blue diamonds) experiences competitive release in a mixed infection with HB3 (black squares) E) No difference is detected between HB3 grown in single culture (open grey squares) and in mixed culture with 7C424 (black squares): F) No difference is detected between 7C424 grown in single culture (open light blue diamonds) and in mixed culture with HB3 (dark blue diamonds). When drug pressure is initiated 120 hours after the start of the assay **(G-I)**: G) 7c424 is competitively suppressed in a mixed infection containing HB3 (black squares) and 7C424 (blue diamonds): H) No difference is detected between HB3 grown in single culture (grey squares) and in mixed culture with 7C424 (black squares). I) 7C424 reaches greater densities when grown in single culture (light blue diamonds) than in mixed culture with HB3 (dark blue diamonds). Initiation of drug pressure is indicated by a black arrow. Parasite densities are shown in genomes per microlitre of culture. Points are means of three replicates, error bars represent standard deviation.

To test for the affects of drug treatment on competition, assays were carried out both in the presence and absence of drugs. Because Dd2 competitively suppressed HB3 in the absence of drugs, we also use a parasite combination for which the CQS (HB3) parasite outgrew the CQR (7C424) parasite in the absence of drugs. Pilot data showed the HB3 outcompeted the CQR clone 7C424 in the absence of drugs; subsequently, drug treated competition assays were carried out using the CQS clone HB3 and the CQR clone 7C424 to test the effect of drug treatment on infections containing more than one parasite. Control assays containing no drug demonstrated that HB3 reached significantly higher densities than 7C424 (*t*-test, *p* < 0.0001) (Figure 2A). The density of HB3 grown in single culture is not statistically different from that grown in mixed culture (Figure 2B). However, 7C424 was suppressed by HB3, i.e. it was found at significantly higher densities in single culture than in mixed culture (*t*-test, *p* = 0.0047) (Figure 2C). Similar to results between Dd2 and HB3, the maximum density reached by 7C424 is reduced six-fold by the presence of HB3 in culture.

Having established that the CQS parasite HB3 competitively suppressed the CQR clone 7C424, the effect of CQ on the dynamics of these two parasites grown together was determined. Parasites were cultured for 72 hours to establish an infection before constant CQ (50 nM) was initiated and maintained for the duration of the assay. Fifty nM lies between the CQ IC_50_ values of HB3 and 7C424 (27.4 nM and 327.5 nM respectively) [19]. Application of drug treatment was delayed to mimic conditions in a mouse model where competitive release was observed when drug treatment was initiated after an infection had been established [17]. Results of the drug treated competition differed markedly from the no-drug control as the resistant clone (7C424) expanded in numbers as the HB3 was cleared by CQ pressure which resulted in a higher density of 7C424 than HB3 (*t*-test, *p* = 0.0008) (Figure 2D). The application of drug not only reversed the outcome of the competition (Figure 2A and 2D) but also resulted in a more pronounced difference in densities between the two parasites. In the no-drug treatment, HB3 was present at a seven-fold higher density, and after drug treatment 7C424 was at 167-fold greater density than HB3. The lower density of HB3 was due to drug pressure and not competitive suppression by 7C424, because HB3 grew similarly alone and in mixed culture (Figure 2E). To determine if treatment-induced facilitation occurred, a comparison was made between the performance 7C424 with and without the application of drugs. If competitive release occurred a difference in parasite growth would be seen with and without the application of drugs. No significant difference was detected between 7C424 grown alone and in mixed culture; the addition of drug pressure effectively removed the competitive suppression by HB3 and led to the competitive release of 7C424, however, the extreme release phenomenon of treatment-induced facilitation (higher numbers in mixed culture than when grown alone) was not observed (Figure 2F).

### Timing of drug treatment affects growth relationships

**Figure 3:**
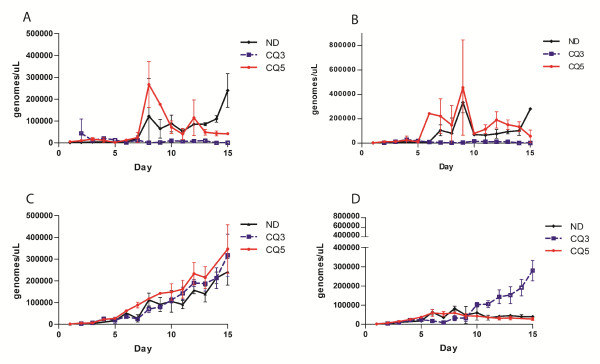
**Results of all drug treated assays. A)** HB3 grown in single culture and **B)** mixed co-cultured with 7C424 in no-drug (black triangles),72 hour drug treated (blue squares) and 120 hour drug treated competition assays (red circles). **C)** 7C424 grown in single culture and **D)** co-cultured with HB3 in no- drug (black triangles), day 3 drug treated (blue squares) and day 5 drug treated competition assays (red circles). Parasite densities are plotted in genomes per microlitre of culture. Points represent means of three replicates. Error bars represent standard deviation.

To investigate the effect of dosing schedules on parasite dynamics, drug pressure was initiated at a later timepoint. Parasites were allowed to grow for 120 hours before the initiation of drug treatment. The density of HB3 at the time of drug treatment (9,198 genomes per microlitre) in the later application is not statistically different from the density at the time of early application (11,860 genomes per microlitre) of drug treatment. The later application of drug allowed time for HB3 to competitively suppress 7C424. The results observed after delayed exposure to drug more closely resembled parasite growth in no-drug controls, as HB3 significantly out-numbered 7C424 (*t*-test, *p* = 0.0069) (Figure 2G and Figure 3). 7C424 is suppressed by HB3 when drug pressure is initiated later as evidenced by a higher density of 7C424 grown alone than when co-cultured with HB3 (*t*-test, *p* = 0.0125) (Figure 2F). These results suggested that, at least in an *in vitro* system, there is only a limited window of time in which drug resistant parasites can be competitively released. The results of all drug treated competition assays are summarized in Figure 3. Future work will focus on defining the boundaries for the timing of drug treatment that leads to competitive release.

## Discussion

The impact of co-infection by more than one genetically distinct *Plasmodium* parasite is an area of increasing interest. Known sequence polymorphisms were used to design allele specific primers to monitor the dynamics of mixed cultures of clones Dd2 and HB3 along with the effect of drug treatment. Dd2 competitively suppressed HB3 in the absence of drug pressure. HB3 competitively suppressed 7C424, a CQR clone, in the absence of drug pressure, however, the application of drug pressure led to competitive release when CQ was applied early but not when drug was applied later. These data represent the first attempt to use absolute quantification of competitive suppression and the effects of drug treatment over time in mixed *P. falciparum* infections *in vitro.* This method provides a useful tool in the monitoring of mixed infections and spread of drug resistant parasites. It is of particular interest that competitive suppression is asymmetrical: Dd2 competitive suppressed HB3, however, HB3 did not effect Dd2 growth. Asymmetrical competition was also observed when HB3 was co-cultured with the CQR clone 7C424 in the absence of drug pressure. HB3 competitively suppressed 7C424; however, the presence of 7C424 had no effect on HB3, consistent with the observations of sensitive parasites suppressing resistant parasites in the absence of drugs observed *in vivo*[17,18]. Testing of further parasite pairs will determine if this dynamics is typical of all mixed culture infections or if it varies by specific parasite combinations.

Comparison of the growth of HB3 and 7C424, when each was suppressed in mixed culture, demonstrated that the shapes of the suppression growth curves were markedly different than their growth curves when grown alone. In fact, the maximum parasite density of HB3 and 7C424 was reduced in mixed culture 10-fold and six-fold respectively. This is an intriguing result because differences in parasite density between parasites grown alone and in mixed culture would be expected to be minimal without parasite interactions; however, in this case it appears that the majority parasite may be inhibiting the growth of the suppressed parasite. These interactions could be as simple as interference competition, wherein a particular clone is able to acquire necessary resources faster; this could also entail a more complex biology such as active competition, wherein one parasite directly inhibits another, as has been reported for bacterial systems [1].

Recently the idea that individual cells in a bacterial population act as autonomous units has been replaced by the hypothesis that social interactions are common in prokaryotic organisms [21]. This phenomenon, known as quorum sensing, involves the accumulation of a signal molecule leading to the activation of signal transduction cascades that repress or activate target genes, bringing about a change in collective behaviour [22]. Although less well studied, evidence of quorum sensing has been observed in eukaryotes [21] and in *Plasmodium*[23-25]. While the data presented here provide no direct evidence for interference competition or quorum sensing, the assay developed here is well suited to study a range of possible interactions, including possible quorum sensing.

Previous studies of mixed infections of human malaria parasites have been somewhat restricted because they determine only relative densities of parasites and because direct evidence of parasites interactions within a human host are difficult to obtain due to ethical reasons. The rodent malaria system allows for the investigation of questions not easily answered using an *in vivo P. falciparum* system. One of the most important questions that can be addressed by quantitative study of rodent malaria is how the presence of more than one parasite can affect the development and spread of drug resistant parasites. It has been argued that when multi-clone infections are common, within-host ecology can be the primary determinate of the speed at which resistance spreads [9]. Two of the most troubling observations concerning the effect of drug treatment on mixed cultures are the phenomena of competitive release and treatment-induced facilitation. Wargo *et al. *[17] found in a rodent model that when a mixed infection containing a drug resistant and drug sensitive clone is treated with drug, the removal of the sensitive parasite, which in the absence of drug competitively suppresses the drug resistant clone, leads to competitive release and allows for the expansion of the drug resistant parasite. The increase in drug resistant parasites is seen both in the asexual stages circulating in the bloodstream and in the transmission stages. Further studies found that the degree of competitive release is dependent on the dose of drug given [18]. Treatment-induced facilitation also has been observed in humans. A study on the effects of intermittent preventive treatment with sulphadoxine-pyrimethamine on pregnant women in Malawi revealed that women who had been subjected to recent drug treatment harboured a less diverse parasite population, likely due to the elimination of drug sensitive parasites by drug treatment [6]. Furthermore, women who had received recent drug treatment were found to have a significantly higher parasitaemia and increased frequency of drug resistance phenotypes, an observation attributed to the competitive facilitation of drug resistant parasites [6]. Potential explanations exist to explain treatment-induced facilitation [6,17]. One is resource competition, i.e. interference competition, between parasites. In this case, removal of the drug sensitive parasites will allow the resistant parasites access to resources that were previously unavailable. An alternative explanation is an immune-mediated response: if the host immune system targets the majority parasite within an infection, the removal of the majority parasite (in this case the drug sensitive) by drug treatment will cause the immune system to then target the minority parasite. If a lag exists in immune retargeting the minority parasite can take advantage and greatly expand in numbers.

Some insight into the mechanism leading to competitive facilitation can be gained from the data in this study. In a mixed infection that is drug treated 72 hours after the initiation of the assay, the resistant parasite population expands as the competitive suppression from the sensitive clone is removed. Unlike previous results [6,17], the density of the drug resistant parasite from the mixed culture does not exceed the density of the resistant parasite grown alone, indicating that the removal of competitive suppression allows the initial release of the resistant parasites. However, the lack of treatment-induced facilitation *in vitro*, where resistant parasite densities in mixed infection exceed that of single infections [17,18], does not rule out the role of an immune mediated host response. Additionally, it is possible that the treatment-induced facilitation phenotype only exists between specific combinations of parasites. Further studies using different combinations of parasites will reveal whether the degree of competitive release observed is genotype dependent. If genotypes are found to have an impact on the magnitude of competitive release, it will be possible to uncover underlying controlling genetic factors. This study also demonstrated that changes in the timing of the initiation of drug treatment had a dramatic effect on the outcome of the competition. When CQ pressure was applied early, the competitive suppression of 7C424 by HB3 was removed and the drug resistant parasite expanded to similar levels observed when grown in single culture; however, when drug treatment was started one cell cycle later, the results were dramatically different. Even though the HB3 parasites were eventually cleared from the culture, they remained able to competitively suppress 7C424. A possible explanation for the difference is a limiting supply of red blood cells; most or all of the red blood cells suitable for parasite invasion may have been used by the time HB3 was eliminated from the culture by drug. If this is the case, 7C424 was therefore unable to expand due to the paucity of available red blood cells, consistent with results observed in *P. chabaudi*[26]. While the number of available red blood cells was not determined following drug treatment to eliminate HB3, such quantification will be possible in future studies using this experimental system. An active suppression mechanism may also be employed by HB3. If this mechanism is similar to those observed in bacteria, a soluble factor in the culture could be indicated. It is possible that a certain number of HB3 parasites must be present for the soluble factor to surpass a threshold density and for this effect to occur, but further studies will be needed to investigate this possibility.

There has been an increased interest in applying mathematical models to predict the effects drug treatment may have on multiple malaria infections. As an example modeling was recently used to determine selection coefficients in drug treated multiple infections of *P. chabaudi*[18]. Models can also offer some insight into the types of interactions that take place within a multiple infection [27]. However these models are reliant on high quality measurement of parasite parameters to make accurate predictions. The methods described here provide an opportunity to quantify the growth of individual parasites both grown alone and within a multiple infection.

The timing and dosage of drug intervention and dosage has been shown to play a significant role in the outcome of mixed infections [17,18]. The assay described here will facilitate the investigation of the effects of various drugs and doses on mixed cultures. If these assays accurately mimic the conditions within a human host, the effects of the timing of drug could have important implications for the malaria control policies to limit the spread of drug resistant parasites.

## Conclusions

Through the development of an absolute quantification competition assay, it was determined that competitive suppression is present when two genetically distinct *P. falciparum* strains are co-cultured *in vitro*. Further, the application of drug to a multiple infection containing both drug sensitive and drug resistant parasites can lead to the removal of competitive suppression, the competitive release of and a greatly increased density of drug resistant parasites. However, the timing of drug application can have a great effect on parasite densities. The tools developed here will enable experiments to dissect interactions between multiple parasite clones in *in vitro* infections with and without drug treatments. This knowledge could inform novel control strategies to contain the spread of drug resistant parasites.

## Competing interest

The authors declare that they have no competing interests.

## Authors’ contributions

MAW and MTF conceived and designed the experiments. MAW, LBT, LAW and MCM performed the experiments and analysed the data. MAW, LBT and MTF wrote the manuscript. All authors read and approved the final manuscript.

## Supplementary Material

Additional file 1 **Standard curves of all clones A) Dd2 DNA alone (black circles) produces clear standard curve (R**^**2**^** = 0.999) and adding HB3 DNA (red squares) does not alter this curve (R**^**2**^** = 0.998).** B) HB3 DNA alone (black circles) produces a clear standard curve (R^2^ = 0.999) and neither the addition of Dd2 DNA (red squares) nor 7C424 DNA (blue diamonds) significantly alters the curve (R^2^ = 0.999, and 0.932 respectively). C) 7C424 DNA alone (blue diamonds) produces a clear standard curve (R^2^ = 0.999) and adding HB3 DNA (black squares) does not significantly alter the curve (R^**2**^ = 0.997). Click here for file
