# Supplementary figures and images for: Quantification of multiple infections of Plasmodium falciparum in vitro
Source: Malar J. 2012 May 30;11:180. doi: 10.1186/1475-2875-11-180 (PMC3483182; doi:10.1186/1475-2875-11-180)

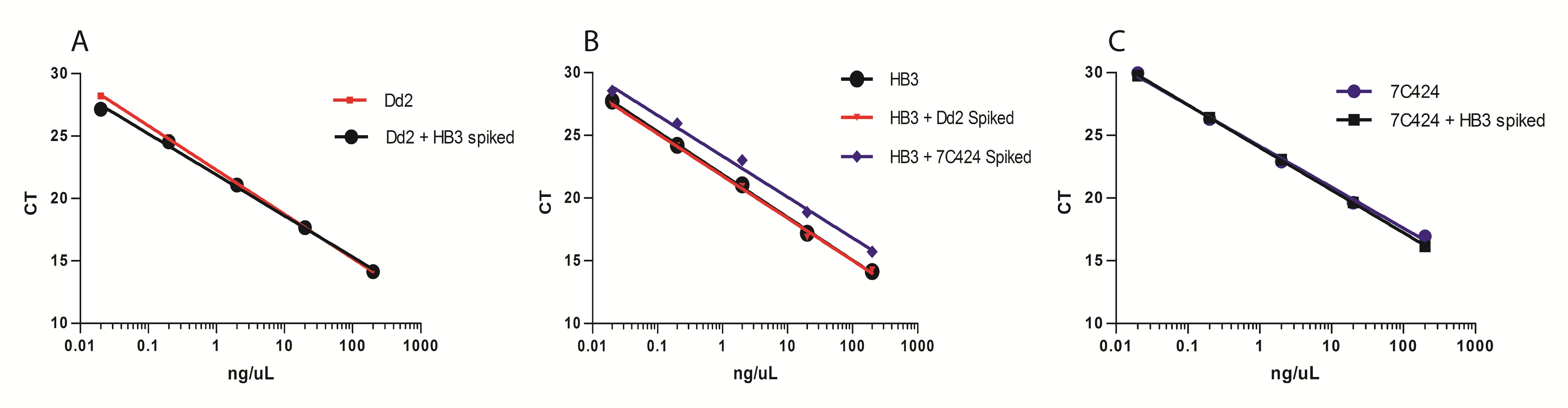

Supplement: Additional file 1 — Standard curves of all clones A) Dd2 DNA alone (black circles) produces clear standard curve (R2 = 0.999) and adding HB3 DNA (red squares) does not alter this curve (R2 = 0.998). B) HB3 DNA alone (black circles) produces a clear standard curve (R2 = 0.999) and neither the addition of Dd2 DNA (red squares) nor 7C424 DNA (blue diamonds) significantly alters the curve (R2 = 0.999, and 0.932 respectively). C) 7C424 DNA alone (blue diamonds) produces a clear standard curve (R2 = 0.999) and adding HB3 DNA (black squares) does not significantly alter the curve (R2 = 0.997). [file 1475-2875-11-180-S1.jpeg]
